# Temporary anchorage devices and the forces and effects on the dentition and surrounding structures during orthodontic treatment: a scoping review

**DOI:** 10.1093/ejo/cjac072

**Published:** 2023-05-31

**Authors:** Colin Ritchie, Scott McGregor, David R Bearn

**Affiliations:** Orthodontic Department, Dundee Dental Hospital and Research School, University of Dundee, Scotland; Library & Learning Centre, University of Dundee, Scotland; Orthodontic Department, University of Dundee, Scotland

## Abstract

**Background:**

Temporary anchorage devices (TADs) offer the clinician an immediate temporary source of skeletal anchorage for a range of orthodontic interventions. It is important to understand forces involved in using TADs and the effects on the dentition and surrounding structures, to improve clinical outcomes.

**Objective:**

To examine and qualitatively synthesize literature on the forces involved with the use of TADs and the effects on the dentition and surrounding structures in orthodontic tooth movement, to provide better understanding of the complex interactions and the clinical implications.

**Search methods:**

Electronic databases searched included: Cochrane Library [including Central Register of Controlled Trials (CENTRAL)], Embase via OVID, Pubmed, and Scopus. Study screening and selection were conducted in duplicate.

**Selection criteria:**

Studies selected were clinical studies, simulation studies (computer or laboratory-based), or animal studies with no restriction over gender, age, study type (excluding case reports), or setting. Studies focusing on the forces involved with the use of TADs in orthodontic treatment and their effects on the dentition and surrounding structures were included.

**Data collection and analysis:**

A data charting form was piloted and refined. Data charting was performed independently and in duplicate. This consisted of key fields with predetermined options and free text. The extracted data were collated, and a narrative synthesis conducted.

**Results:**

The results from 203 included studies were grouped into seven TAD based interventions combining the clinical, simulation, and animal studies. They were: En masse retraction of anterior teeth, intrusion, movement of a single tooth, orthopaedic interventions, distalisation, maxillary expansion and other types. The forces involved with the use of TADs, and their effects on the dentition and surrounding structures, were presented in descriptive and tabular formats.

**Limitations:**

This review restricted study language to English. Formal appraisal of the quality of evidence is not a required feature of scoping reviews, as per the PRISMA-ScR guidelines, however it was evident that a proportion of clinical studies were of high risk of bias and low quality and therefore any proposed changes the reader may consider to their clinical practice should be contextualized in light of this.

**Conclusions:**

Across the seven types of TAD based interventions the effects on the dentition and surrounding structures are described providing a better understanding of the complex interactions. A guide to the level and direction of forces in each type of intervention is provided to aid clinicians in achieving high quality outcomes.

**Implications:**

There is a need to validate future FEA simulation studies by comparing to clinical data. It is also recommended that future scoping reviews incorporate a formal critical appraisal of studies to facilitate the translation of the results into clinical practice. Development of a standard set of terms for TADs is recommended to facilitate future research.

**Registration:**

Registration of a scoping review is not possible with PROSPERO

**Funding:**

None to declare.

## Introduction

Temporary anchorage device (TAD) use in orthodontics has increased throughout the last ten years (1). TAD is an umbrella term for several appliance types that offer an immediate and temporary source of skeletal anchorage for a wide range of orthodontic tooth movements. The majority of the current TAD based clinical research has focused on the design, stability, failure rates, anchorage effectiveness, and optimal locations for miniscrews, miniplates, and onplants (2–6).

However, TADs introduce a different system of force mechanics into the conventional tooth-based force systems that orthodontists are accustomed to with fixed appliances supported by conventional intra-oral anchorage appliances. The nature of the anchorage and the position of a TAD relative to the fixed appliance and the centre of resistance of the teeth and jaws, creates direct and indirect forces that, on the whole, are not well understood. It is important therefore to recognize that TADs have introduced a new concept in managing orthodontic anchorage and orthodontists need to have an understanding of the forces, and the unwanted and unexpected effects that may be associated with TADs, in order to improve clinical outcomes (1,7,8).

Current systematic reviews of TADs have focused on quantitative outcomes using interventional systematic review methodology, for example reporting amounts of anchorage gained or lost and tooth movements (9,10). To move from this knowledge based quantitative approach to a conceptual understanding of the force systems associated with TADs requires the qualitative approach of a systematic scoping review. A scoping review aims not to report and synthesize quantitative results from randomized clinical trials but instead to take a broad overview of different types of published research on a topic to gain a better conceptual understanding (11).

For example, in addition to clinical studies there is a large body of literature based on computer simulations, in particular Finite Element Analysis (FEA). FEA is an engineering-based computational method to model the stress distribution and associated deformations of structures under loading, and has been applied in biomechanical fields such as orthodontics (12). There is also animal-based literature that can potentially be beneficial in progressing the current understanding of the forces involved and effects of TADs (13).

### Review objective

The objective of this scoping review is to examine and qualitatively synthesize literature focusing on the forces associated with the use of TADs in orthodontic tooth movement, and the effects of those forces on the dentition and surrounding structures, to better understand the anchorage concept associated with TADs. A formal critical appraisal of studies is not a standard feature of scoping review methodology, instead synthesizing information from a wide range of clinical, simulation, and animal studies to yield clinically useful information to facilitate the understanding required for the effective use of TADs.

### Review question

The scoping review mnemonic PCC (Population, Concept, Context) was utilized to develop a review question, being the equivalent to the PICOS (Population, Intervention, Comparison, Outcome, Study design) mnemonic used in interventional systematic reviews (14).

The ‘population’ was clinical patients, simulated patients (computer or laboratory-based), or animal models with the use of any type of TAD with no restriction on age. The ‘concept’ was the forces and effects on the dentition and surrounding structures from the use of TADs. The ‘context’ considered all TAD based uses in orthodontic tooth movement in any setting with no restriction on location.

The review question was: ‘What are the documented forces associated with the use of TADs in orthodontic treatment and the effects of those forces on the dentition and surrounding structures?’ 

## Materials and methods

The conduct and reporting of this scoping review followed the Joanna Briggs Institute (JBI) methodology for scoping reviews (14) and the Preferred Reporting Items for Systematic Reviews and Meta-Analyses Extension for Scoping Reviews (PRISMA-ScR) guidelines (15). Registration of a scoping review is not possible with PROSPERO.

### Inclusion criteria

The criteria used to screen studies for inclusion were developed based on the PCC and study type:

#### Population

Studies with clinical patients, simulated patients (computer or laboratory-based), or animal models involving the use of any type of TAD with no restriction over gender or age. The term TAD encompassed all variations for the three main groups of TADs—miniscrews, miniplates, and onplants. Dental implants or surgical screws utilized for the purposes of orthodontic treatment on a temporary basis were also included.

#### Concepts

The use of TADs to generate forces for orthodontic tooth movement and the effects of those forces. The studies included are those that analysed the clinical, biomechanical, radiographic, or histological effect of the forces associated with the use of TADs for orthodontic purposes, on the dentition, bone, or maxillofacial structures.

#### Context

This scoping review considered all TAD based interventions for orthodontic tooth movement in any setting with no restriction on location.

#### Types of sources

This scoping review considered clinical studies (experimental studies, observational studies, and systematic reviews), simulated studies (computer based or laboratory-based), and animal studies. Study language was restricted to English. The clinical experimental designs included uncontrolled prospective interventional studies, controlled trials, and randomized control trials. The clinical observational studies included observational descriptive (case series) or observational analytical studies (case control or cohort).

### Exclusion criteria

The exclusion criteria were:

- Studies focused on the success or failure rates of TADs, or on TAD design (such as size/length/threads) in relation to TAD stability
- Studies that focus on TAD insertion without applying a force to the TAD as part of orthodontic tooth movement or simulated tooth movement
- Studies that describe the use of TADs but do not attempt to measure or scientifically analyse the forces involved, and their effects on dentition, bone, or maxillofacial structures.
- Opinion papers/clinical technique/case reports
- Studies not relating to oral and maxillofacial region
- Studies focused on dental implants for restorative purposes and implant retained prostheses
- Studies focused on physiology/pharmacology
- Studies focused on orthognathic surgery/condyle fractures/maxilla and mandible fractures/clefts.
- Studies focused on pain/discomfort

### Search strategy and implementation

A comprehensive strategy using a combination of controlled vocabulary (MeSH terms) and free text terms was developed to search published, ongoing and unpublished studies (Supplementary Table 1). The search strategy was modified for each database by an information specialist (SM) and one reviewer (CR). There was no restriction on publication dates and the results were limited to studies published in the English language. The search strategy was implemented by the information specialist (SM) on 29 May 2019. Updated searches were performed on 23 July 2020, 31 July 2021, and 5 July 2022 using the same methodology, search strategy, and information sources.

Databases searched electronically were Cochrane Library (including Central Register of Controlled Trials (CENTRAL)), Embase (OVID), PubMed, and Scopus. The Clinical Trial Registry, *ClinicalTrials.gov*, and the grey literature site, Open Grey were also searched. In addition, a hand search was performed in *American Journal of Orthodontics and Dentofacial Orthopedics*, European Journal of Orthodontics, Angle Orthodontist, Journal of Orthodontics, Seminars in Orthodontics, Australian Orthodontic Journal, and the *Korean Journal of Orthodontics*. Reference lists of identified studies and other relevant systematic reviews were manually inspected for additional studies. Google Scholar was also searched. Supplementary Table 2 details the search strategy adapted for PubMed.

### Data collection and analysis

#### Study screening and selection

Following the removal of duplicates, the titles of the articles identified from the searches were assessed independently, and in duplicate, by two reviewers (CR and DB) for eligibility based on the defined inclusion and exclusion criteria. Abstracts of potential articles were then assessed independently, and in duplicate, by two reviewers (CR and DB). The full texts of the articles of relevant and potentially relevant studies, and for studies that appeared to meet the inclusion criteria but for which there was insufficient information in the title and abstract to make a decision, were obtained. The full texts were examined independently, and in duplicate, by CR and DB and assessed for eligibility. Any disagreements were resolved through discussion between CR and DB.

Data charting is the appropriate method for data extraction in scoping reviews. This was performed independently and in duplicate by CR and DB for the original and updated searches using customized forms developed in Microsoft Excel. The form consisted of fields with predetermined options using the drop-down-list functionality or free text boxes. The individual study data charted is shown in Supplementary Table 3. Any disagreements were discussed by CR and DB and resolved through dialogue. The authors of the studies were contacted to clarify existing data, and to request missing data or additional data.

The data charting form aligned with the research objective and question. For training and calibration purposes both reviewers independently charted the first 20 studies and compared and discussed their findings to determine the applicability and robustness of the data charting form. The charting form was refined following this pilot stage. Inter-rater reliability was assessed by calculating percent agreement statistics.

**Figure 1:**
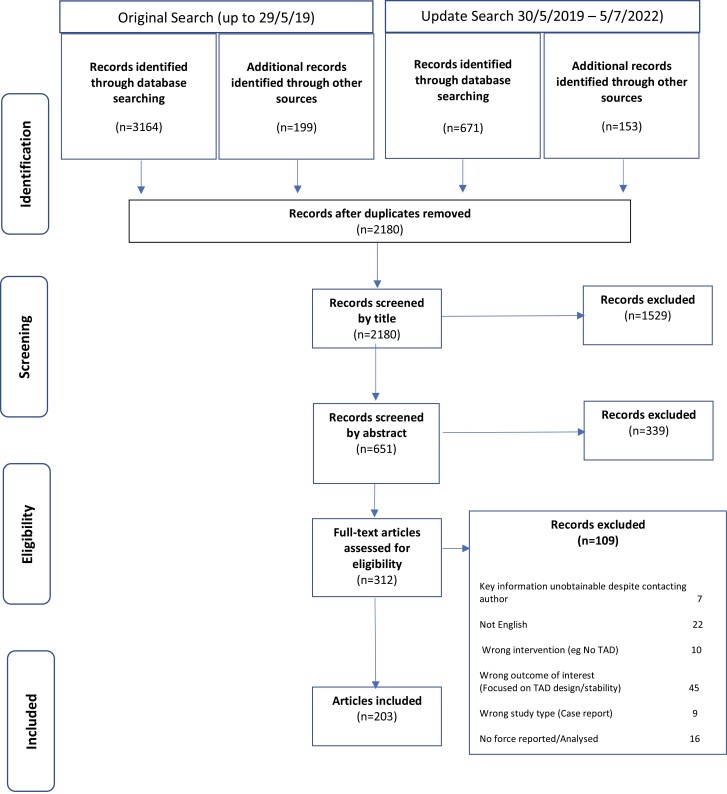
PRISMA-ScR flow chart.

A web-based application, Rayyan (www.rayyan.ai), was used to facilitate the screening process (16). From the group of full text articles, the excluded studies with reasons for exclusion were recorded (Figure 1). Supplementary Table 3 contains a list of the included and excluded studies from the full paper screening stage. 

## Results

### Results of the search

Figure 1 shows the number of studies identified at each stage of the screening process following the original and the updated search processes.

### Characteristics of included studies

#### Characteristics of study location and publication dates

The largest group of studies (*n* = 67, 33%) were conducted in East Asia followed by Europe and South Asia (Table 1).

**Table 1.** Location of the included studies per region.

Region	Number of studies per region
East Asia	67
Europe (including Turkey)	35
South Asia	34
North America	16
Africa	17
South America	15
Middle East	13
South East Asia	6

#### Characteristics of journal of publication

The majority of studies were published in an orthodontic based journal (*n* = 140, 69%) followed by non-orthodontic dental journals (*n* = 26, 13%). Seven studies (3%) were published in an engineering-based journal and 10 studies were registered trials (5%). The remainder were published in other journals.

#### Characteristics of type of TAD used and TAD terminology

To facilitate the data synthesis and reporting process the review authors charted the type of TAD utilized in the studies into three categories, irrespective of the variations of TAD terminology used by the studies’ authors. The review authors made a determination on which category the TAD best suited based on the characteristics reported. These categories were mini screw, mini plate, and onplant.

The majority of studies involved the use of mini screws (*n* = 156, 77%). Twenty-three (11%) studies employed mini plates and four studies (2%) used surgical screws for orthodontic purposes. No study investigated onplants. The remainder used a combination of the above or were unclear. Table 2 shows the variations in TAD terminology.

**Table 2.** Variations in temporary anchorage devices (TAD) terminology used by the authors of the included studies.

TAD terminology (as determined by the authors of this review)	Variations of TAD terminology used in the included studies	Number of studies
Mini screw	Mini screw	79
	Mini implant	44
	Micro implant	8
	Mini screw implant	7
	Implant	7
	Orthodontic Mini Implant (OMI)	3
	Micro screw	4
	Temporary anchorage device (TAD)	2
	Temporary anchorage skeletal device (TASD)	1
	Micro screw implant	1
Mini plate	Mini plate	23
Onplant	Not Studied	Not studied
Surgical screw used for orthodontic purposes	Surgical screw	4
	Study used a combination of the above or was unclear	20

#### Characteristics of the study types and publication dates

The largest group of studies is simulation-based studies, either computer modelling or laboratory-based experiments (*n* = 99, 49%). Eighty-seven (43%) of the studies are clinical studies. Sixteen studies (8%) are animal-based studies and one study was a combination of animal and simulation study types. Of the 87 clinical studies, 24 are controlled or uncontrolled interventional studies, 34 are randomized controlled trials, and 23 are retrospective observational studies. Six were systematic reviews. The first year a study was published was 2001. The year with the largest number of studies published was 2019 (*n* = 33, 16%). Table 3 shows the trends in the numbers of each study type over time.

**Table 3.** Trends in the numbers of each study type over time (note, one study was a combination of animal and simulation types and is not included in this table).

Study population	Date of study publication	Number of studies published
Animal	2000 – 05	1
	2006 – 10	3
	2011 – 15	8
	2016 – 22	4
Clinical	2000 – 05	1
	2006 – 10	13
	2011 – 15	24
	2016 – 22	49
Simulation	2000 – 05	0
	2006 – 10	7
	2011 – 15	35
	2016 – 22	57

#### Characteristics of the clinical samples

Out of the clinical studies seven had sample sizes of ten or less. Thirty-eight studies involved sample sizes of 11 to 29 and 29 studies had 30 to 49 participants. The remaining studies had 50 or more participants. Twenty-three of the clinical studies involved subjects under 18 years of age and 12 studies focused on adults. The remaining studies involved both adults and subjects under 18 years.

#### Characteristics of the simulation and animal samples

Ninety studies involved FEA simulations, six studies used a laboratory typodont setup, one study used a laboratory-based photoelastic setup, and two studies used ‘other’ types of laboratory setup or computer simulation. Out of the animal studies three studies used pigs, nine studies used dogs, three studies used rat models, and one study employed monkeys.

#### Characteristics of the TAD based interventions

There were seven main types of TAD based interventions investigated across the studies. The largest number of studies involved space closing by en masse retraction of anterior teeth (*n* = 50, 24%), followed by intrusion studies (*n* = 39, 19%) and movement of single teeth such as canines (two-step retraction), molar protraction or molar uprighting (*n* = 22, 11%). Orthopaedic interventions and distalisation mechanics were also studied extensively (*n* = 23, 11% and *n* = 20, 10%, respectively). There was a group of studies investigating TAD based rapid maxillary expansion (*n* = 21, 10%). Other types of TAD based interventions involved one study focused on extrusion mechanics and animal studies investigating scenarios involving TADs connected to other TADs (*n* = 4, 2%). Finally, 25 studies (12%) involved a combination of the above. When comparing this data to the publication date data for the last three years, it is evident the current focus is on TADs for en masse retraction of anterior teeth and intrusion.

#### Characteristics of the forces used in the studies

Table 4 shows the range of forces (converted into grams) used for each type of TAD based intervention.

**Table 4.** Forces used for each temporary anchorage device(TAD) intervention across the studies (X—not reported/applicable).

Type of TAD based intervention	Clinical studies	Animal studies	Simulation studies
En masse retraction anterior teeth	100 – 200 g	X	100 – 200 g
Intrusion – anterior	40 – 60 g	40 g	40 – 60 g
Intrusion – posterior	150 – 400 g	150 g	150 – 450 g
Movement of single teeth (such as two-step canine retraction, or molar protraction)	100 – 200 g	3 – 200 g	50 – 1500 g
Orthopaedic Interventions	100 – 500 g	200 g	350 – 1000 g
Distalisation	100 – 400 g	X	150 – 300 g
Maxillary expansion	No forces reported. 1 – 4 turns per day	X	800 – 100 000 g
Other (mainly TAD connected to TAD)	X	150–600 g	X

#### Characteristics of the effects on surrounding structures

The effects on the dentition and surrounding structures of using the TAD based interventions focused mainly on the dentition, bone, periodontal ligament, skeletal relationships, and the facial soft-tissues.

The largest effect investigated by the studies was the effect on the dentition. This mainly involved analysing the stress (von Mises stress) and strain generated in the teeth. The linear and angular tooth displacements, anchorage loss, rates of tooth movement or force moments in all three dimensions, and any associated root resorption, were analysed. The centre of resistance/rotation of teeth or jaws and the effect on the occlusal plane were also analysed by several of the studies.

The next largest effect investigated by the studies was the effects on bone. These studies analysed the cortical and cancellous regions of alveolar bone, facial bones, cranium, or sutures in relation to stress and strain, bone volume/density changes, or bone-TAD contact characteristics on a histological level.

The studies analysing the periodontal ligament mainly focused on the stress and strain within the ligament and histological changes. Studies investigating skeletal relationships assessed the maxillary or mandibular skeletal changes or changes in cranial structures in three dimensions. The effect on facial soft-tissues, mainly naso-labial angle, and facial convexity, were assessed by several studies.

### Synthesis of results

The findings of the studies included in this scoping review were grouped into seven TAD based interventions. A narrative synthesis of the data was conducted in relation to the use of TADs in orthodontic treatment, the forces involved, and the effects on the dentition and surrounding structures.

#### En masse retraction of anterior teeth

The clinical studies involving en masse retraction of anterior teeth mainly investigated the effectiveness of using buccally placed miniscrews in various positions versus conventional anchorage such as molar blocks or transpalatal arches. The consensus is that whilst miniscrew reinforced anchorage may not be any faster, it does produce less unwanted dental and skeletal effects compared to conventional anchorage, such as less mesial and extrusive movements, or tipping and rotation of molars (9,17–27). Several of the clinical studies reported intrusion of molars is possible with en masse retraction depending on the vertical positions of the miniscrew (28). This may be favourable for high anchorage cases with increased vertical proportions.

A group of clinical studies investigated the effect of varying the height of the retraction arm when using miniscrews for en masse retraction (25,28–32). The consensus is that the type of tooth movement can be controlled by changing the height of the retraction arm. It was shown that a retraction arm of three to five mm delivers controlled lingual crown tipping. As the retraction arm is lengthened, bodily movement and intrusion of the anterior teeth can be achieved with less palatal tipping of the incisors and less rotation of the maxillary occlusal plane occurring, which is beneficial for correcting cases with deep bite and increased gingival exposure. This is due to the retraction force vector passing closer to the centre of resistance of the anterior teeth (30). Several of the clinical studies have demonstrated beneficial soft-tissue effects such as improvements in facial convexity, naso-labial angle, and upper and lower lip protrusion, mainly due to the increased control of the anterior and posterior teeth (9,25,27,32–34).

Simulation studies investigated miniscrews placed in a high, medium, or low vertical position, approximately 13 mm, 10 mm, and 7 mm above the archwire, respectively. This was usually between the 2nd premolar and 1st molar (35–38). Using an anterior retraction arm of approximately 9 mm with the medium miniscrew position delivered bodily movement of the anterior teeth as the retraction vector is closer to the centre of resistance (35,38–42). This agrees with the findings from the clinical studies.

The simulation studies also demonstrated a high miniscrew position with a shorter retraction arm will cause molar intrusion and retraction of the anterior teeth and an anti-clockwise rotation of the occlusal plane (38,43–46). A low miniscrew position will cause retraction and palatal tipping and a clockwise rotation of the occlusal plane especially with a shorter retraction arm. Several studies have shown that as the retraction arm is lengthened, such that the incisor teeth intrude, canine extrusion may occur due to a downward deflection of the archwire during incisor intrusion (43,44).

A group of simulation studies investigated the location of the centre of resistance of the six maxillary anterior teeth. The consensus is approximately 9 mm superiorly and 13 mm posteriorly from the midpoint of the central incisor crown. The centre of resistance of the entire maxillary dentition is estimated to be 4 mm above the alveolar crest at the second premolar level (35,47,48).

The simulation studies also showed that as extraction spaces close, the resultant force vector changes relative to the centre of resistance, resulting in different anterior tooth movement and occlusal plane rotations. Therefore, the force system delivered during treatment should be regularly visualized and modified to suit the current teeth position in relation to the centre of resistance (44). Modifications to the archwire such as compensating curves and using a full sized archwire can help compensate for any unwanted incisor movement (44).

Several simulation studies showed the optimal insertion angle of a miniscrew for retraction mechanics is 90 degrees to the bone surface which minimizes the magnitude of stress in the bone (48–51). These studies propose that by keeping the bone stress levels within physiological limits, this will improve the miniscrew stability. Consolidating the maxillary anterior teeth using a ligature wire reduces unwanted labial flaring, facilitates more effective retraction and reduces stress in the teeth, PDL, bone, and miniscrew (50,51).

##### Summary

Overall, the literature on en masse retraction of anterior teeth using TAD anchorage shows a more complex balance of factors than orthodontists are familiar with when using molar anchorage alone (with or without conventional anchorage support). The variations in TAD placement position and retraction arm length, open the opportunity for more appropriate tooth movements to be achieved for optimal outcome in individual cases if this complex interaction is understood, but also the risk of unwanted results with occlusal plane rotation if inappropriate forces are generated.

#### Intrusion

The clinical studies investigating the effects of anterior and posterior intrusion mainly involved various combinations of miniscrews with sectional or continuous archwires.

For effective intrusion of the maxillary central and lateral incisor teeth the evidence converged on using a single miniscrew in the region between the central incisors below the anterior nasal spine. The miniscrew would be connected to the archwire via power chain providing approximately 50 g of force. In contrast to conventional systems involving intrusion arches, this system of mechanics delivers true intrusion with minimal labial or palatal tipping and avoids reactionary effects on the molar teeth such as molar extrusion (52–60).

Using two anterior miniscrews, between the upper canine and lateral incisor teeth, will deliver greater canine intrusion as the force vectors act closer to the canines (56,61). However, this arrangement can cause palatal tipping of the maxillary incisors which may or may not be favourable depending on the malocclusion. Posteriorly positioned miniscrews between the second premolars and first molars can be connected to an anterior sectional archwire to deliver anterior intrusion and retraction simultaneously (54). Compared to anteriorly placed TADs, this arrangement produces labial crown tipping of the anterior teeth which may be favourable depending on the starting incisor inclination, and causes less root resorption (54).

For posterior intrusion in anterior open bite cases, it has been shown that buccal miniscrews used in conjunction with a transpalatal arch, or zygomatic miniplates used with a rapid maxillary expansion (RME) appliance, is effective (62). The latter study involving the zygomatic miniplates and the RME appliance reported approximately 4 mm of molar intrusion without unwanted buccal tipping of the molars (62). The mandible autorotates anti-clockwise due to the intrusion of the upper molars (62,63).

Clinical studies evaluating molar intrusion, using buccal and palatal miniscrews simultaneously, showed that effective molar intrusion is possible especially in patients with a hyperdivergent skeletal pattern (64,65). It was suggested that a difference in bone density between hyperdivergent and hypodivergent patterns was a contributing factor (64).

Other clinical studies reported no difference in intrusion effectiveness or root resorption between 200 g and 400 g intrusion force when using infrazygomatic palatal or buccal miniscrews to intrude buccal segments for anterior open bite cases (66,67).

The simulation-based studies mainly investigated the stresses generated by using miniscrews for intrusion. It was shown that stresses in the bone surrounding a non-osseointegrated miniscrew are higher than if it was osseointegrated, but the increased stress was still within physiological limits of bone (68). The highest bone stresses are found in the apical and palatal region of anterior teeth and the furcation area of posterior teeth (69–72). It has been demonstrated that by using three posterior TADs (two buccal and one palatal) and a transpalatal arch, the stresses generated by posterior molar intrusion are reduced (73). Additional techniques such as corticotomy can reduce the bone stress (74).

One simulation study showed that greater maxillary molar intrusion is achieved by placing the miniscrew more posteriorly (between the 1st molar and 2nd molar) whilst minimizing undesirable anterior intrusion or labial tipping. By using a transpalatal arch, tipping of posterior teeth was also minimized which correlates with the results of the clinical studies (69,70). It was shown that the counterbalancing effect of using buccal and palatal TADs produces a balanced set of force mechanics which is conducive with true intrusion without unwanted tipping (72,75,76).

Simulation studies also showed that mandibular anterior teeth can be intruded, for example by placing miniscrews between the lower canine and 1st premolar bilaterally, and using two sections of power chain on each side in a triangular arrangement between the miniscrews and the archwire (77). This produces true intrusion as the resultant force system is balanced and is close to the centre of resistance of the mandibular anterior teeth (77).

Four animal studies investigated the histological changes in the bone surrounding the TAD, and root resorption, and found that intrusion mechanics caused limited root resorption however, this was greater when the root was impacted against cortical bone (78–81). Forty grams of continuous force is appropriate for incisor intrusion which correlates with the clinical studies. Approximately 150 g of force is appropriate for molar intrusion.

##### Summary

Overall, the literature on intrusion using TADs supports the effectiveness of this technique for anterior and posterior intrusion but highlights the risk of flaring of the teeth. For upper anterior intrusion placement of the TAD is critical to obtain the desired clinical effect as counterbalancing forces are difficult to generate. The most effective location is a single TAD placed labially in the midline with a force level below 50 g. For posterior intrusion the counterbalancing force from buccal TADs causing flaring can be from auxiliary appliances such as a TPA or RME appliance or the use of palatal TADs, with a force of 150–200 g.

#### Movement of a single tooth

There were eight clinical studies investigating movement of a single tooth—mainly canine retraction (two-step retraction in contrast to en masse retraction) or molar protraction or uprighting. Four of these studies involved surgical and non-surgical adjunctive procedures aimed at accelerating tooth movement—corticotomy, vibration, piezocision, and micro-osteoperforation—concluding that adjunctive procedures produced acceleration of tooth movement (27,82–85). The remaining four studies compared TAD based retraction of canines to conventional anchorage procedures (86–89). The conclusions were that TAD based anchorage reduced the amount of anchorage loss or unwanted movements of anchorage teeth and that direct sliding mechanics between the TAD and tooth bracket itself is faster than using a power arm, however not using a power arm may introduce unwanted anterior tipping movements.

There were eight simulation studies using FEA and one laboratory-based typodont model investigating the bone stress induced by molar uprighting, canine retraction, and molar protraction (90–98). The highest level of stress was found to be in the cortical bone as opposed to cancellous bone but remained within physiological levels. Bone stress can be reduced by using a longer retraction arm when retracting canines. Miniscrews produced the least bone stress when uprighting second molars compared to using opencoil, T-loops, or cantilever springs.

Six animal studies investigated the bone reaction and healing around a TAD by connecting TADs to animal teeth or connecting to a second TAD, and applying a force (90,99–103). Immediate loading does not significantly affect the bone volume or healing, and light forces are favourable. This corroborates the findings of the simulation studies, that despite bone stresses being greater when miniscrews are not osseointegrated, the stresses are not high enough to cause miniscrew failure. These studies therefore suggest that miniscrews can be loaded immediately.

#### Orthopaedic interventions

Twelve clinical studies involved orthopaedic change in patients with maxillary deficiency. Eight of these clinical studies used miniplates and four used miniscrews (104–115). The majority involved the use of TADs directly or indirectly attached to a Petit type facemask. One study involved a Forsus Class 2 corrector appliance with TADs and the remainder used TADs only, placed in the maxilla and mandible, to deliver inter-maxillary traction. Across these twelve clinical studies the TADs were placed either anteriorly in the lateral incisor region, posteriorly between the second premolar and first molar, in the zygomatic buttress regions or on the palate. There was agreement across these studies that using TADs delivers greater maxillary advancement compared with non-TAD based protraction interventions, with less unwanted effects such as incisor proclination or retroclination and less occlusal plane rotation.

The simulation-based studies using FEA investigated the stresses generated in the maxilla and amount of maxillary advancement associated with TAD based protraction (116–120). Similar to the clinical studies, TADs were used directly or indirectly with a facemask. The angle of the protraction force vector varied between 15 degrees and 55 degrees in a downward direction.

Across the simulation studies, TADs were placed in various positions including the lateral nasal wall region, infrazygomatic crest, or palate. The position of the TADs and resulting force vector relative to the centre of resistance of the maxilla determine the amount of maxillary advancement and its direction. For a patient with average vertical proportions, a force vector of 30 degrees in a downward direction is optimal for translatory movement of the maxilla with minimal occlusal plane rotation. For brachyfacial patients, a force vector at 45 degrees in a downward direction is recommended, and for dolichofacial patients, a force vector at 15 degrees in a downward direction is suitable. Less bone stress and unwanted dental effects, such as incisor inclination change or molar tipping, is associated with TAD based protraction methods.

Two animal studies showed that inter-maxillary traction using miniplates placed posteriorly in the maxilla, and anteriorly in the mandible, produced significant maxillary advancement compared to conventional protraction methods (121,122). Histologically, greater bone stress tension was observed within the facial sutures with TAD based inter-maxillary traction producing greater maxillary advancement.

#### Distalisation

Eleven clinical studies examined distalisation of upper molar teeth in Class II patients (123–133). These studies involved either directly attaching a posteriorly positioned TAD to the archwire in the canine region or using the TAD indirectly by incorporating it into a distalising appliance such as the Pendulum appliance, the Dual Force Distaliser, iPANDA, or a modified transpalatal arch. In general, these studies demonstrated that using TAD based interventions are more effective with less unwanted effects such as maxillary incisor protrusion.

During distalisation, molar intrusion can be controlled by altering the number of TADs used and their position. It was demonstrated that four maxillary TADs used simultaneously, one between the 2nd premolar and 1st molar and the other between the two premolars bilaterally, creates a resultant force that passes close to the centre of resistance of the maxilla, which is in the premolar region (126). This arrangement delivers whole arch distalisation and intrusion and minimizes clockwise rotation of the occlusal plane that can occur when using a single TAD bilaterally.

Eight simulation studies using FEA assessed the effect of vertical positioning of the TAD in relation to the point of force application on the archwire (134–141). TAD position included buccally between the 1st molar and 2nd premolar, the retromolar region and palatally. For maxillary molar distalisation a force application nearer the archwire produced less vertical effects. To achieve bodily molar distalisation in the mandible, whilst minimizing vertical movement, the resultant force vector should be approximately parallel to the occlusal plane and at the cemento-enamel junction level.

#### Maxillary expansion

Nine clinical studies investigated maxillary expansion (104,142–149). Various designs of tooth-borne or bone-borne expanders incorporating miniscrews were compared to similar expanders without miniscrews. The majority of these studies agreed that greater maxillary expansion is achievable by incorporating miniscrews with less unwanted effects such as buccal tipping of molar teeth. It was shown that miniscrew assisted rapid maxillary expansion (MARME) is more effective than traditional rapid maxillary expansion during both pre-pubertal and post-pubertal development stages. Several of these studies reported MARME can cause the maxilla to rotate downwards and forwards during expansion and the facial soft-tissues can be displaced forward and laterally.

The simulation studies involved the use of miniscrews incorporated into various expansion appliances (150–156). These mainly assessed the stresses associated with various MARME mechanics. MARME produced relatively even stress distribution in the mid-palatal suture region and was effective in opening the suture including in adult patients. There was less stress on the buccal plates and less molar tipping. The antero-posterior position of the miniscrews did not significantly affect the expansion achieved however it does impact on the vertical maxillary displacement. Anteriorly positioned palatal miniscrews caused relatively more clockwise rotation of the maxilla therefore reducing the antero-posterior change.

##### Summary

Overall, MARME has been shown to be more effective than conventional RME techniques with a more even stress distribution across the maxillary structures leading to less unwanted side-effects. Again, the importance of understanding the effect of different TAD positioning on the outcomes, in this case in relation to maxillary rotation and displacement is highlighted.

#### Other types of TAD based interventions

Four animal studies explored the effects of immediate loading of TADs on bone (103,157–159). The studies connected TADs to other TADs with active components such as elastomeric powerchain. Following loading, there was an increase in bone density, hardness and bone volume surrounding the TAD. The forces involved ranged from 50 g to 300 g. This suggests that immediate loading of TADs stimulates a favourable physiological response. Taken into consideration with the studies on single tooth movement, there is consensus across the range of different experimental models included here, that immediate loading of TADs is appropriate.

## Discussion

This scoping review has included a diverse body of literature across clinical, simulation, and animal studies in relation to the utilization of TADs in orthodontic treatment, the associated forces, and the effects on the dentition and surrounding structures to aid better understanding of the concept of anchorage that TADs provide. The approach taken is systematic following the Joanna Briggs Institute (JBI) manual for scoping reviews (14) and has been reported in line with the Preferred Reporting Items for Systematic Reviews and Meta-Analyses Extension for Scoping Reviews (PRISMA-ScR) (15). We recognize that this approach is very different from the interventional systematic review that is common in the orthodontic literature, and which focuses on quantitative assessment and synthesis through meta-analysis.

The recent increase in interest in qualitative methods and patient related outcomes in orthodontics (160,161) is in line with the qualitative approach that is core to the scoping review methodology (15,161). In this process, a broad sweep of the literature is reviewed in a systematic manner to give better understanding of key or new concepts. It enables conclusions to be drawn from across different research methodologies and this is particularly relevant in TADs where there is a large amount of simulation literature that sheds light on clinical outcomes and informs clinical decision making but is not included in the more familiar interventional review focussing on randomized clinical trials. Bringing these aspects together with animal research findings helps to build a better understanding of the TAD anchorage concept and how to deliver high quality patient outcomes. It is evident from this review that the use of TADs can produce favourable and unfavorable effects however, the literature supports that TADs offer more advantages than non-TAD based anchorage.

A recurring theme in the results is the direct relationship between the centre of resistance of the dentition and the maxillary/mandibular arches, and the line of action generated between the TADs and point of force application. The position of the TAD in three dimensions and the point of application will generate force vectors that can cause occlusal plane rotations and effect the dentition, skeletal relationships, and facial soft-tissues such as the naso-labial angle. In order for the clinician to alter the TAD position and number, and select a point of application to achieve the desired tooth movement and minimize unwanted effects, an understanding of the complex interactions described here is essential. Orthodontists should consider these when planning TAD placement and the related biomechanics to mitigate any potential adverse outcomes. It should also be recognized that the system of force mechanics will change as tooth movement ensues, and therefore this should be regularly reviewed throughout treatment.

This review has investigated the force application for seven TAD based orthodontic interventions across clinical, simulation, and animal studies aiming to give better understanding and so guide clinicians in delivering appropriate force systems for effective orthodontic tooth movement whilst remaining within physiologic limits of bone.

The amount of FEA simulation literature addressing this review’s question has been increasing steadily and is now the most common study method adopted by the global scientific community. En masse retraction of anterior teeth and intrusion using TADs are currently the two most common concepts being researched, particularly in East Asia.

The variation in TAD terminology across all the studies in this review was significant especially for the miniscrew category. This dichotomy of terms can potentially limit the effectiveness of literature searches and impede the synthesis of data. It is recommended that a standard set of terms for TADs is developed and adhered to thereby facilitating researchers and clinical readers.

### Study limitations

This review restricted study language to English which may potentially limit the completeness of the results. Formal appraisal of the quality of evidence is not a required feature of scoping reviews, as per the PRISMA-ScR guidelines (15), however it was evident that a proportion of clinical studies were of high risk of bias and low quality and therefore any proposed changes the reader may consider to their clinical practice, based on the results of this review, should be contextualized in light of this. It is recommended that future scoping reviews consider the appropriateness of incorporating a formal critical appraisal of studies.

One important characteristic of simulation studies is the ability to replicate orthodontic scenarios, measure forces with ease, and optimize the force mechanics to produce the desired outcome. However, a limitation of FEA studies is that to be effectual it is crucial that the simulations accurately represent the biological systems they are replicating. There is a gap in the literature on validation of FEA models in orthodontics directly comparing the findings to matched clinical situations to show validity. This is an area that should be addressed by future studies.

## Conclusion

The results of this scoping review have reported on seven types of TAD based interventions and the effects on the dentition and surrounding structures, providing a better understanding of the complex interactions and has provided a guide to the level and direction of forces in each type of intervention to aid clinicians in achieving high quality outcomes.

### Implications of the findings for research

There is a need to validate future FEA simulation studies by comparing to clinical data. It is also recommended that future scoping reviews incorporate a formal critical appraisal of studies to facilitate the translation of the results into clinical practice. Development of a standard set of terms for TADs is recommended to facilitate future research.

## Supplementary Material

cjac072_suppl_Supplementary_Table_1

cjac072_suppl_Supplementary_Table_2

cjac072_suppl_Supplementary_Table_3

## Data Availability

*The data underlying this article are available in the article and in its online supplementary materials.*
