# Supplementary material for: Temporary anchorage devices and the forces and effects on the dentition and surrounding structures during orthodontic treatment: a scoping review
Source: Eur J Orthod. 2023 May 31;45(3):324–37. doi: 10.1093/ejo/cjac072 (PMC10230247; doi:10.1093/ejo/cjac072)
Supplement: cjac072_suppl_Supplementary_Table_1 [file cjac072_suppl_supplementary_table_1.docx]

**Supplementary Table 1: Comprehensive Search Terms based on the PCC Framework.**

Boolean Operators ‘OR’ used
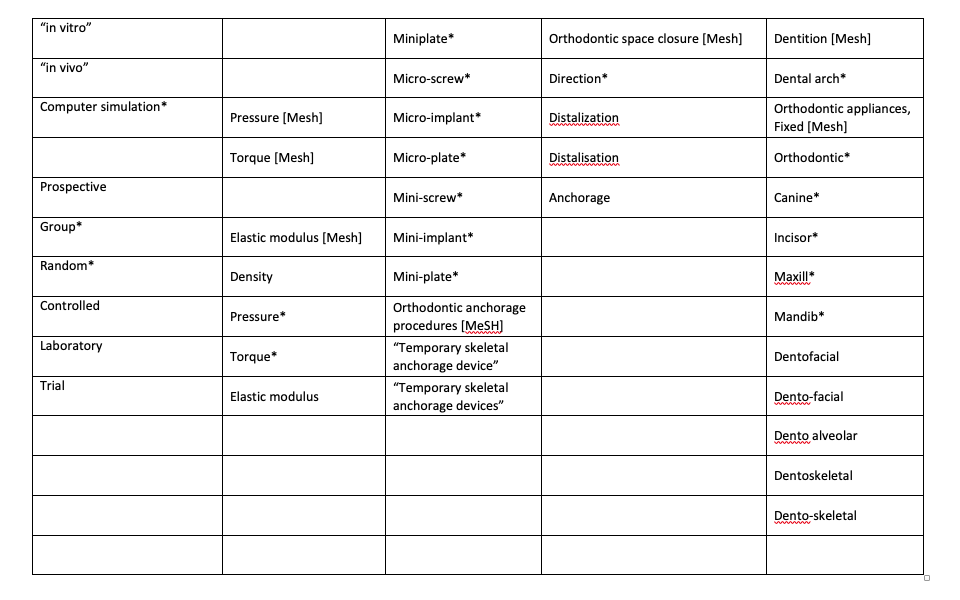

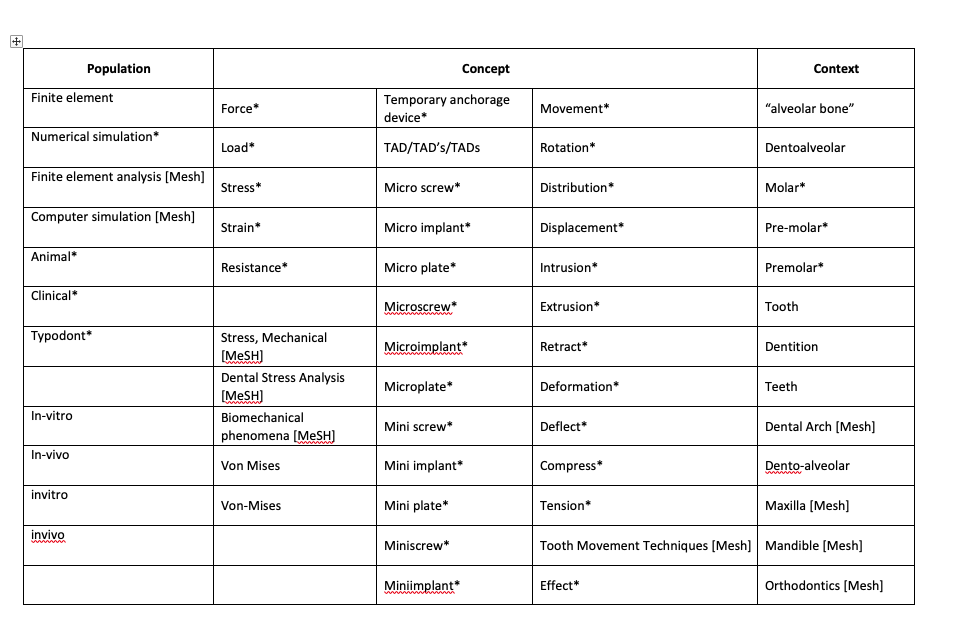
between rows, ‘AND’ between columns
