# Supplementary material for: Temporary anchorage devices and the forces and effects on the dentition and surrounding structures during orthodontic treatment: a scoping review
Source: Eur J Orthod. 2023 May 31;45(3):324–37. doi: 10.1093/ejo/cjac072 (PMC10230247; doi:10.1093/ejo/cjac072)
Supplement: cjac072_suppl_Supplementary_Table_2 [file cjac072_suppl_supplementary_table_2.docx]

**Supplementary Table 2: Search Strategy Adapted for Pubmed**

**Search strategy for Pubmed**

(((((((((((((((((((((((((((((((“alveolar bone”) OR Dentoalveolar) OR Molar*) OR Pre-molar*) OR Premolar*) OR Tooth) OR Dentition) OR Teeth) OR "Dental Arch"[Mesh]) OR Dento-alveolar) OR "Maxilla"[Mesh]) OR "Mandible"[Mesh]) OR "Orthodontics"[Mesh]) OR "Dentition"[Mesh]) OR Dental arch*) OR "Orthodontic Appliances, Fixed"[Mesh]) OR Orthodontic*) OR Canine*) OR Incisor*) OR Maxill*) OR Mandib*) OR Dentofacial) OR Dento-facial) OR Dento alveolar) OR Dentoskeletal OR Dento-skeletal)) AND ((((((((((((((((((Movement*) OR Rotation*) OR Distribution*) OR Displacement*) OR Intrusion*) OR Extrusion*) OR Retract*) OR Deformation*) OR Deflect*) OR compress*) OR tension*) OR "Tooth Movement Techniques"[Mesh]) OR effect*) OR "Orthodontic Space Closure"[Mesh]) OR Direction*) OR Distalization) OR Distalisation) OR Anchorage)) AND (((((((((((((((((((((((((Temporary anchorage device*) OR TADs) OR TAD) OR TAD's) OR Micro screw*) OR Micro implant*) OR Micro plate*) OR Microscrew*) OR Microimplant*) OR Microplate*) OR Mini screw*) OR Mini implant*) OR Mini plate*) OR Miniscrew*) OR Miniimplant*) OR Miniplate*) OR Micro-screw*) OR Micro-implant*) OR Micro-plate*) OR Mini-screw*) OR Mini-implant*) OR Mini-plate*) OR "Orthodontic Anchorage Procedures"[Mesh]) OR “Temporary skeletal anchorage device”) OR “Temporary skeletal anchorage devices”)) AND (((((((((((((((((Force*) OR Load*) OR Stress*) OR Strain*) OR Resistance*) OR "Stress, Mechanical"[Mesh]) OR "Dental Stress Analysis"[Mesh]) OR "Biomechanical Phenomena"[Mesh]) OR Von Mises) OR Von-Mises) OR "Pressure"[Mesh]) OR "Torque"[Mesh]) OR "Elastic Modulus"[Mesh]) OR Density) OR Pressure*) OR Torque*) OR Elastic modulus))) AND ((((((((((((((((((((Finite element) OR Numerical simulation*) OR "Finite Element Analysis"[Mesh]) OR "Computer Simulation"[Mesh]) OR Animal*) OR Clinical*) OR Typodont*) OR In-vitro) OR In-vivo) OR invitro) OR invivo) OR “in vitro”) OR “in vivo”) OR Computer simulation*) OR Prospective) OR Group*) OR Random*) OR Controlled) OR Laboratory) OR Trial)
